# Changes in adipokine levels and metabolic profiles following bariatric surgery

**DOI:** 10.1186/s12902-022-00942-7

**Published:** 2022-02-03

**Authors:** Natalja Šebunova, Jelena Štšepetova, Tiiu Kullisaar, Kadri Suija, Anneli Rätsep, Igor Junkin, Hiie Soeorg, Margus Lember, Toomas Sillakivi, Reet Mändar

**Affiliations:** 1 Department of Microbiology, Institute of Biomedicine and Translational Medicine, University of Tartu, Ravila street 19, 50411 Tartu, Estonia; 2 Competence Centre on Health Technologies, Tartu, Estonia; 3 Department of Biochemistry, Institute of Biomedicine and Translational Medicine, University of Tartu, Tartu, Estonia; 4 Department of Family Medicine, Faculty of Medicine, Institute of Family Medicine and Public Health, University of Tartu, Tartu, Estonia; 5 Family Doctors Takker ja Sarapuu Ldt, Tartu, Estonia; 6 Department of Internal Medicine, Faculty of Medicine, Institute of Clinical Medicine, University of Tartu, Tartu, Estonia; 7 Department of Internal Medicine, Tartu University Hospital, Tartu, Estonia; 8 Abdominal Surgery Department, Tartu University Hospital, Tartu, Estonia; 9 Department of Surgery, Faculty of Medicine, Institute of Clinical Medicine, University of Tartu, Tartu, Estonia

**Keywords:** Adipokines, Bariatric surgery, Metabolic syndrome, Weight loss, Blood indices

## Abstract

**Background:**

Bariatric surgery is considered to be the most effective treatment option for weight reduction in obese patients. Abdominal obesity is frequently accompanied by metabolic syndrome (MS). Adipokines are cell signaling proteins that have direct impact upon the metabolic homeostasis. The purpose of this analysis was to evaluate the effect of bariatric surgery, including laparoscopic sleeve gastrectomy (LSG) and laparoscopic gastric bypass (LRYGB) on the adipokine levels and metabolic profile as well as MS and status of type 2 diabetes (T2D).

**Methods:**

We analyzed anthropometric parameters, blood levels of adipokines, vitamins, lipids and inflammatory markers in 30 bariatric surgery patients with obesity of class II or III 1 month before and 1 year after surgery as well as in 60 obese patients from general practice (GP) and 15 patients with normal body mass (control).

**Results:**

The BMI was significantly higher among patients before surgery and GP patients in comparison to control and post-surgery patients. The levels of glucose, cholesterol and LDL-cholesterol, triglyceride and hs-CRP were the highest in patients before surgery but decreased significantly after surgery, while the level of HDL-cholesterol increased after surgery.

The levels of adiponectin increased and that of leptin decreased after surgery. The significant difference in the concentration of resistin was revealed between LSG and LRYGB methods. The relationship between resistin and vitamin D was also found. The patients with MS and T2D displayed significantly greater reduction in lipid markers and adipokine levels than the rest of patients.

**Conclusion:**

Remarkable changes in levels of adipokines after bariatric surgery appear like increase in adiponectin and decrease in leptin levels. Significant improvement in anthropometric parameters, metabolic and inflammatory markers occurs, suggesting high potential for reduction of metabolic syndrome and risk for type 2 diabetes. We have shown for the first time ever that level of vitamin D may be involved in resistin regulation.

**Supplementary Information:**

The online version contains supplementary material available at 10.1186/s12902-022-00942-7.

## Background

Overweight and obesity belong to the main public healthcare problems in many developed countries. The causes of obesity are considered to be the combination of genetic, epigenetic and environmental factors [1–3]. The prevalence of overweight and obesity in Estonia has reached up to 35.0% and 32.0% respectively [4, 5].

In case of morbid obesity, the human organism suffers from an excess of subcutaneous and visceral adipose tissue. Adipokines are the factors produced by adipose tissue. The most important of them are leptin, resistin and adiponectin [6]. Abnormal shifts in adipokine levels are associated with obesity-related diseases such as insulin resistance, type 2 diabetes (T2D), hyperlipidemia, stroke, atherosclerosis and different types of cancer [7–15].

A few methods are available for management of severe obesity, bariatric surgery being the most effective [16, 17]. Laparoscopic sleeve gastrectomy (LSG) and laparoscopic gastric bypass (LRYGB) are the most commonly performed bariatric surgery procedures [18]. Studies have shown that bariatric procedure may correct metabolic comorbidities, regardless of weight loss [19–21] while less is known about shifts in adipokine levels after bariatric surgery. Since increasing number of people becomes eligible for bariatric surgical treatment, we should have a better understanding of how this procedure may influence the markers of health, including adipokine levels which are important in the pathogenesis of obesity and metabolic comorbidities.

The aim of the study was to reveal the impact of different bariatric surgery methods on the levels of the most relevant adipokines. We also aimed to assess the relationship of adipokines with anthropometric and blood parameters and its changes in patients with metabolic syndrome (MS) and type II diabetes (T2D) in consequence of bariatric surgery.

## Materials and methods

### Study groups

The study comprised three cohorts of patients. The bariatric surgery group (BS group) was formed of 30 consecutive morbidly obese bariatric surgery patients with class II or III obesity during the period March 2015 – October 2018 at the Tartu University Hospital. This group included 19 females and 11 males with the mean age 46.9±8.9 (range 26-61) years and BMI 44.3±5.6 (range 37-64) kg/m^2^, being investigated 1 month before and 1 year after surgery. These patients underwent bariatric surgery using one of the two methods: laparoscopic gastric bypass (LRYGB, *n*=12) or laparoscopic sleeve gastrectomy (LSG, *n*=18). All patients were operated by the same surgical team. The GP group was recruited during the period April 2015 – May 2018 by general practitioners working in family practitioner centers in Tartu (Ülikooli Perearstikeskus OÜ), Elva (Elva Kesklinna Perearstikeskus OÜ) and Kambja (Kambja Perearsikeskus OÜ), it consisted of consecutive volunteers (20 females and 40 males) with mean age 50.9±10.2 (range 29-75) years and mean BMI 36.0±4.4 (range 29.2-52.6) kg/m^2^. The control group (C group) consisted of 15 consecutive volunteers recruited from the family practitioner center in Tartu (Perearstid Takker ja Sarapuu OÜ) and included 9 females and 6 males; mean age 35.8 ±9.2 (range 28-54); years mean BMI 22.9±2.2 (range 19-26) kg/m^2^ . Basic clinical parameters of all study subjects are presented in Table 1 and in Supplementary table 1.

**Table 1:** The values of anthropometric and blood variables in study subjects

Indices	Reference values mean±SD (range)	Mean±SD [median (25th and 75th percentile)]				*P*-value
		Obese patients in GP practice (*n*=60)	Obese patients going to bariatric surgery (*n*=30)		Control group (*n*=15)	
			Before surgery	1 year after surgery		
Age (years)						
Total		50.9±10.2 [50.5(43-58)]^c^	46.9±8.9 [48.5(41-53)]^a^	48.1±8.9 [49.5(42-54.3)]^b^	35.8±9.2 [37(28-42)]^a,b,c^	^a,b,c^ *p*<0.001
Male		51.8±10.5 [53(43.3-58.8)]^a^	45.5±6.5 [47(41-5)]^b^	46.7±6.5 [48(42-53)]^c^	30±9.72 [27.5(22.5-37.3)]^a,b,c^	^a,b,c^ *p*<0.001
Female		49.3±9.6 [47.5(41.5-6)]^a^	47.7±10.1 [50(40-56)]^b^	49±10.1 [52(42-57)]^c^	39.7± 6.9 [40(36-42)]^a,b,c^	^a^ *p*=0.01 ^b,c^ *p*=0.02
Weight (kg)		105.8±14.5 [104.2(95.0-117.1)]^b,d,f^	129.1±22.9 [125.0(112.8-142.8)]^a,b,c^	86.4±18.9 [82.0(73.3-93.3)]^a,d,e^	70.1±10.5 [69.0(63.0-76.0)]^c,e,f^	^a*,b*,c*,d*,f^ *p*<0.001 ^e^ *p*=0.001
BMI (kg/m^2^)						
Total	<18.5 – underweight 18.5-24.9 – normal ≥25 – overweight 25.0-29.9 – pre-obese 30.0-34.9 – obese class 1 35.0-39.9 – obese class 2 ≥40 – obese class 3	35.9±4.4 [35.2(32.7-38.2)]^b,d,f^	44.3±5.6 [44.0(40.0-47.0)]^a,b,c^	29.5±4.6 [28.5(26.0-32.0)]^a,d,e^	22.9±2.2 [23.0(21.0-24.0)]^c,e,f^	a*,b*,c*,d*,e*,f* *p*<0.001
Male		37.0±4.6 [36.4(33.7-39.9)]^a,b,c^	44.5±5.3 [44.0(40.0-50.0)]^a,d,e^	29.9±5.9 [28.0(26.0-35.0)]^b,d,f^	22.2±2.6 [22.0(19.8-24.5)]^c,e,f^	^a,b,c,d,e^ *p*<0.001 ^f^ *p*=0.008
Female		34.0±3.3 [34.1(31.8-36.9)]^a,b,c^	44.4±5.8 [44.0(40.0-46.0)]^a,d,e^	29.3±3.9 [30.0(26.0-32.0)]^b,d,f^	23.3±1.9 [24.0(22.0-24.5)]^c,e,f^	^a,b,c,d,e,f^ *p*<0.001
hs-CRP (mg/l)	<5.0	3.9±4.3 [2.3(0.9-6.4)]^b,d^	6.0±5.4 [4.0(2.0-6.3)]^a,b,c^	1.2±0.6 [1.0(1.0-1.0)]^a,d^	1.3±0.7 [1.0(1.0-1.5)]^c^	^a*,c*^ *p*<0.001 ^b*,d*^ *p*=0.02
Cholesterol (mmol/l)	<5.0	4.6±1.1 [5.6(4.9-6.3)]^b^	5.1±1.0 [5.1(4.4-5.6)]^a^	4.5±0.6 [4.6(4.1-4.8)]^a,b,c^	5.1±1.0 [5.2(4.1-5.2)]^c^	^a^ *p*=0.004 ^b^ *p*<0.001 ^c^ *p*=0.04
LDL–cholesterol (mmol/l)	<3.0	3.5±1.0 [3.4(3.1-4.2)]^b^	3.4±1.0 [3.1(2.8-4.1)]^a^	2.6±0.5 [2.7(2.2-2.9)]^a,b,c^	3.6±0.7 [3.8(2.9-4.1)]^c^	^a*,b,c^ *p*<0.001
HDL–cholesterol (mmol/l)	>1.0	1.4±0.3 [1.4(1.1-1.6)]^b,c^	1.4±0.7 [1.2(1.1-1.4)]^a^	1.8±0.4 [1.8(1.5-2.2)]^a,b^	1.6±0.5 [1.5(1.2-1.8)]^c^	^a*,b^ *p*<0.001 ^c^ *p*=0.025
Triglycerides (mmol/l)	<1.7	1.9±0.2 [1.4(1.1-2.5)]^c,d^	1.8±0.9 [1.8(1.2-21.3)]^a,b^	1.1±0.5 [1.0(0.7-1.5)]^a,c^	1.2±0.7 [0.9(0.8-1.2)]^b,d^	^a*,c*^ *p*<0.001 ^b*^ *p*=0.005 ^d*^ *p*=0.007
Glucose (mmol/l)	4.1-6.1	5.8±0.9 [5.8(5.2-6.1)]^b,d,e^	6.7±2.3 [5.9(5.4-6.8)]^a,b,c^	5.2±1.1 [4.9(4.7-5.3)]^a,d^	4.8±0.7 [5.0(4.3-5.4)]^c,e^	^a,b,c,d,e^ *p*<0.001
A1C (%)	4.0-6.0	5.7±0.4 [5.6(5.4-5.8)]^b,d,f^	6.0±1.3 [5.7(5.5-6.0)]^a,b,c^	5.3±0.4 [5.2(5.1-5.5)]^a,d,e^	5.0±0.3 [5.1(4.8-5.2)]^c,e,f^	^a, b, c, d*,f*^ *p*<0.001 ^e*^ *p*=0.03
A1C (IFCC) (mmol/l)	20.0-42.0	38.3±4.3 [37.7(35.5-39.9)]^c,e^	42.3±13.0 [39.0 (37.0-42.0)]^a,b^	34.5±4.7 [33.0(32.0-36.5)]^a,c,d^	31.1±2.6 [32.0(28.5-33.0)]^b,d,e^	^a*,b*,c*,e*^ *p*<0.001 ^d^ *p*=0.04
Vitamin D (25-OH) (nmol/l)	>75.0	48.8±16.9 [47.9(36.1-58.7)]^c^	54.2±14.7 [54.1(40.7-63.0)]^a,b^	67.9±21.6 [69.3(48.6-80.8)]^a,c^	81.8±75.5 [60.2(40.0-83.5)]^b^	^a^ p =0.006 ^b,c^ *p*<0.001
Vitamin B12 (pmol/l)	145.0-569.0	ND	304.4±108.1 [297.0(234.3-354.5)]	297.8±119.1 [277.0(210.0-387.0)]	337.8±134.4 [327.0(209.5-471.5)]	NS
Vitamin B9 (nmol/l)	8.8-60.8	ND	15.3±7.5 [13.7(10.7-20.3)]^a^	28.9±23.6 [20.4(11.4-36.7)]^a^	15.1±6.5 [13.1(10.9-16.5)]	^a^* *p*=0.03
Ferritin (μg/l)						
Male	30.0–400.0	ND	202.3±82.9 [245.1(127.0-262.0)]	192.8±89.5 [176.8(116.8-241.7)]	226.3±86.7 [203.7(157.7-317.7)]	NS
Female	13.0–150.0	ND	110.4±66.6 [84.0(60.8-154.2)]	114.1±89.7 [78.6(34.8-203.2)]	87.1±90.7 [47.9(40.2-153.6)]	NS
Fe (μmol/l)						
Male	10.6-28.3	ND	19.1±5.7 [17.8(13.9-25.1)]	22.3±6.6 [20.2(17.3-25.1)]	18.9±3.2 [19.2(15.8-21.7)]	NS
Female	6.6-26.0	ND	16.2±6.0 [15.0(12.6-18.7)]	20.6±9.3 [19.2(15.1-24.1)]	17.7±3.8 [18.5(14.9-20.2)]	NS

*BMI* body mass index, *hsCRP* C-reactive protein, *A1C* glycated hemoglobin; *LDL-cholesterol* low density cholesterol, *HDL-cholesterol* high density cholesterol, *ND* not detected, *NS* not significant; Fe-iron

^*^Mann Whitney Sum Rank Test

The patients were divided into subgroups based on their clinical data in eHL system (eHealth) – patients with (MS+) and without metabolic syndrome (MS-), as well as patients with diabetes (T2D+) and without diabetes (T2D-). Metabolic syndrome was diagnosed if three or more of the following five criteria were present: waist circumference ≥102 cm for men and 88 cm for women; blood triglyceride levels ≥1.7 mmol/l; HDL-cholesterol levels ≤1.0 mmol/l in men and 1.3 mmol/l in women; systolic blood pressure ≥130 mmHg or diastolic blood pressure ≥85 mmHg; blood glucose levels ≥5.6 mmol/l [22]. The MS+ group included 58 patients while MS- group included 25 patients (Supplementary table 3). The T2D+ and T2D- subgroups of BS patients consisted of 22 and 8 patients respectively (Supplementary table 4).

The present study was conducted according to the guidelines laid down in the Helsinki Declaration. The study was approved by the Ethics Review Committee on Human Research of Tartu University, Estonia (protocol no 244/T-13). Participation in the study was voluntary. Written informed consent was obtained from all study subjects.

### Anthropometric measurements and blood indices

Anthropometric measurements and blood samples in BS patients were taken in bariatric surgery office of Tartu University Hospital 1 month prior to and 1 year after surgery. In the rest of patients these measurements and samples were taken during routine GP consultation. Body weight was measured to the nearest 0.1 kg using a calibrated manual weighing scale. Height was measured to the nearest 0.1 cm on a standardized wall-mounted height board. BMI was defined as weight in kilograms divided by height in meters squared (kg/m^2^).

Blood samples were obtained in the morning after fasting. Samples were drawn from the antecubital vein with a vacutainer into heparinized tubes and stored at 4°C. The levels of plasma glucose (Glucose), glycated hemoglobin (A1C), lipids: total cholesterol (cholesterol), low density cholesterol (LDL-cholesterol), high density cholesterol (HDL-cholesterol), triglycerides (Triglycerides), high-sensitivity C-reactive protein (hs-CRP) and vitamins D, B12 and B9, ferritin and iron (Fe) were analyzed by standard laboratory methods using certified assay in the United Laboratory of the Tartu University Hospital. The levels of vitamins B12, B9, ferritin and Fe were measured in bariatric surgery patients and the control group.

### Measurement of adipokines

The levels of adipokines (adiponectin, leptin, resistin) in blood serum were analysed by commercially available Quantikine® ELISA kits (R&D Systems, Minneapolis, USA) according to manufacturer protocol: Human total Adiponectin/Acrp30 Immunoassay kit, Human Leptin Immunoassay, Human Resistin* Immunoassay. The reference ranges of adipokines were presented in manufacturer’s protocols.

### Statistical analysis

Statistical analyses were performed using the statistical package SigmaPlot 12.0 (Systat Software Inc., San Jose, California, USA). The differences in anthropometric and blood parameters including adipokines between the study groups were analysed using Mann-Whitney rank sum test, the Wilcoxon signed-rank test and t-test according to the data distribution. *P*-value s less than 0.05 were considered significant. Correlations between changes of variables were explored using Spearman and Pearson’s correlations. Adiponectin, leptin and resistin levels were calculated using Graph Pad Prism version 4.0 (GraphPad Software, San Diego, California, USA). Also, we used MANOVA method to analyse associations between adipokines and surgery methods.

## Results

### Clinical background data

The baseline characteristics for each study group are presented in Table 1 and Supplementary table 1. The body weight and BMI were significantly higher before surgery and among GP practice patients in comparison to the control group and BS group after bariatric surgery. Male patients were heavier before surgery than female patients (147.0±24.1 *vs*. 118.7±14.7 kg, *p*=0.03), however, their mean BMI was quite similar. The weight loss between males and females after bariatric surgery was not significantly different.

The glucose level was the highest in patients before surgery in comparison to other study groups and significantly decreased after bariatric surgery (*p*<0.001, Table 1). The mean levels of glycated hemoglobin were in normal values for all study groups, however, there was increased level of this marker in 8 patients from GP practice (range 42.1-49.7 mmol/l), in 5 patients before surgery group (range 44-95 mmol/l) and in 1 patient after bariatric surgery (50 mmol/l). The levels of glycated hemoglobin also decreased after surgery (*p*=0.006, Table 1).

The mean levels of cholesterol were quite similar in all study groups, being near upper level of the normal value but significantly decreased after bariatric surgery (5.1±0.9 *vs*. 4.5±0.6 mmol/l, *p*=0.004, Table 1). The level of LDL-cholesterol also significantly decreased after bariatric surgery (3.4±1.0 *vs.* 2.6±0.5 mmol/l, *p*<0.001). The level of triglycerides was remarkably higher in patients before surgery and obese patients in GP practice in comparison to the control group and the patients after surgery (*p*<0.01 for all comparisons). In opposite, the level of HDL-cholesterol increased after bariatric surgery (*p*<0.001), although all study subjects featured normal values of this marker.

The level of hs-CRP was notably higher in obese patients before surgery in comparison to GP and control group patients (*p*=0.02 and *p*<0.001) and decreased after bariatric surgery (6.0±5.4 *vs*. 1.2±0.5 mg/l, *p*<0.001). The level of vitamin D in blood samples was the highest in control group and the lowest in obese patients of GP practice and significantly increased too after bariatric surgery (*p*=0.006, Table 1).

### Adipokines

**Fig. 1:**
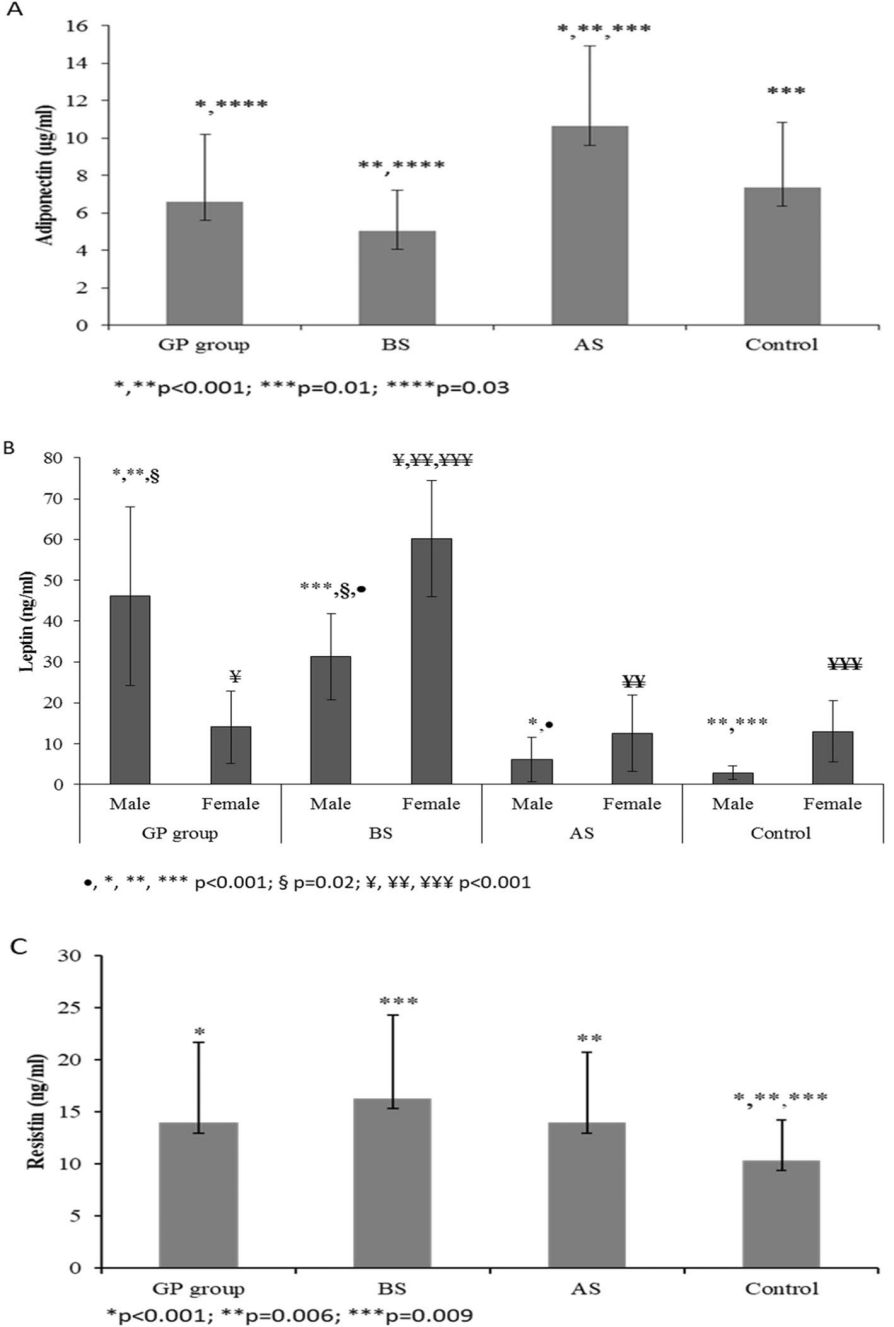
Baseline plasma levels of adipokines among different groups of patients. Bar graphs show plasma levels of adiponectin (**A**), leptin (**B**) and resistin (**C**) among patients with obesity before and after bariatric surgery and control patients. Values represent mean±SD. The x-axis represents the groupings of patients according to study groups, the y-axis represents plasma concentration of relative adipokines. Numerical values are presented in Supplemenatary Table S2

All patients had adiponectin blood value in normal range before and after surgery. The lowest level of adiponectin was detected in obese patients before surgery compared with patients from GP practice and after surgery (5.1±2.2 *vs*. 6.9±3.6 and 10.6±4.3μg/ml, *p*=0.03). Postoperatively, its level almost doubled. Interestingly, postoperative level of adiponectin was even higher in comparison with the control group (Fig. 1A, Supplementary table 2).

Regarding leptin, different reference values for healthy males and females were proposed (Human Leptin Immunoassay, R&D Systems, Minneapolis, USA). Before surgery, the level of leptin was higher than normal level in 11 males and one female. The post-surgery level of leptin was higher than normal range in 3 males and lower in 4 males and one female. The highest levels of leptin in both males and females were observed in obese patients and decreased after bariatric surgery (males: 31.3±10.5 *vs.* 6.1±5.5, *p*<0.001; females: 60.2±14.3 *vs.* 12.5±9.4 ng/ml, *p*<0.001, respectively). In the BS group and control group, the leptin levels in males were lower than in females. In the GP group, the levels of leptin were quite low in females being normal in all cases (Fig. 1B, Supplementary table 2).

For resistin, the lowest levels were obtained in the control group being remarkably different from BS and GP groups (10.9±3.9 ng/ml vs. 16.3±8.0 ng/ml and 14.0±7.8 ng/ml; *p*=0.009, *p*<0.001, respectively). Before surgery, two patients had increased, and one had decreased level of resistin compared to normal range. The decrease tendency was noted after surgery, though it was statistically insignificant (Fig. 1C, Supplementary table 2).

### Impact of surgery method on levels of adipokines

**Fig. 2:**
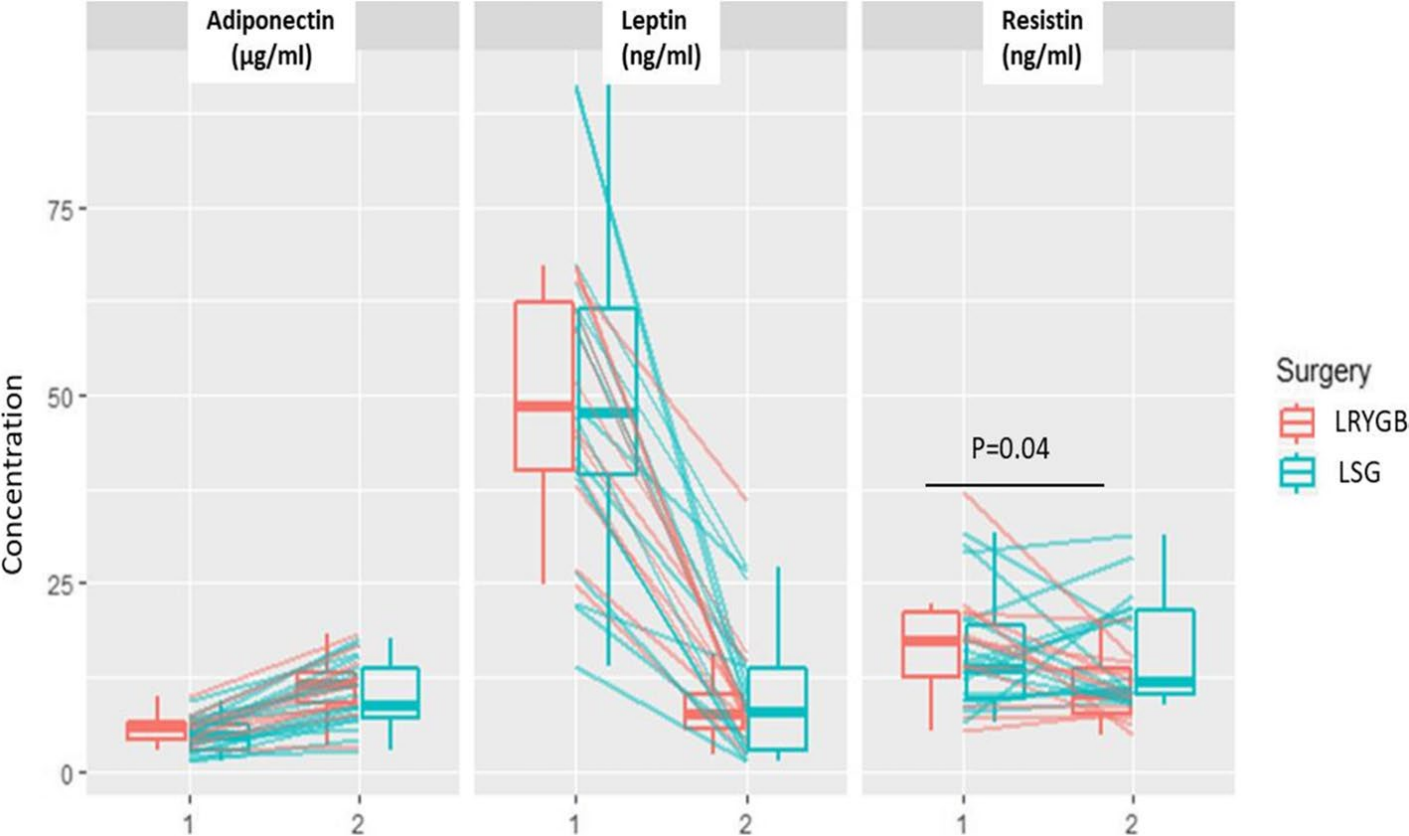
The changes of adipokines levels according to bariatric surgery methods: (LRYGB-red) gastric bypass and (LSG-blue) sleeve gastrectomy. Boxplots represent median plasma levels of adiponectin, leptin and resistin among patients with obesity before and after bariatric surgery. The boxes represent the 25th and 75th percentiles, whereas the bars represent minimum and maximum values. Lines represent changes in adipokines values. The y-axis represents plasma value of relative adipokines, x-axis represents the number of visits: before (1) and after surgery (2)

Comparisons of plasma levels of adiponectin, leptin and resistin between two surgery methods (LSG and LRYGB*)* were also performed. We found difference between two bariatric surgery methods only in case of resistin – the mean value decreased in case of LRYGB by 6.18±7.51 ng/ml while in case of LGS by 0.24±9.21 ng/ml, *p*=0.04 (Fig. 2).

### Correlation between adipokine levels and clinical characteristics

**Fig. 3:**
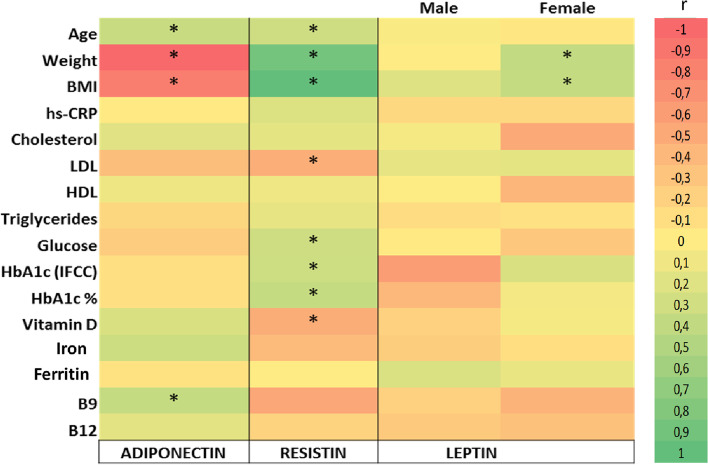
Heatmap indicating Spearman correlation (r) between adipokines' levels and clinical biomarkers of all study groups. Statistically significant correlations (p < 0.05) are indicated with asterisk. The coefficient in each cell ranges from − 1 to 1. A negative value denotes a negative correlation (red), a positive value denotes a positive correlation (green), 1 indicates a complete correlation, and 0 indicates no correlation

The correlations between adipokines and anthropometric and metabolic markers are shown in Fig. 3. The level of adiponectin displayed positive correlations with age (r^2^=0.229; *p*=0.007) and vitamin B9 (r^2^=0.2241; *p*=0.049), and negative correlations with weight (r^2^=-0.385; *p*<0.001) and BMI (r^2^=-0.319; *p*<0.001). The level of leptin correlated positively with weight (r^2^=0.198; *p*=0.04) and BMI (r^2^=0.251; *p*=0.05) in females. The level of resistin was notably associated with age (r^2^=0.203; *p*=0.01), weight (r^2^=0.551; *p*<0.001) and BMI (r^2^=0.620; *p*<0.001) while weak negative association was seen in case of LDL-cholesterol (r^2^=-0.174; *p*=0.05) and vitamin D (r^2^=-0.175; *p*=0.04).

### Comparison of obese patients with and without metabolic syndrome

No significant differences in serum levels of adipokines were found among all patients with and without MS (Supplementary table 3). MS-patients had lower weight and BMI than MS+ patients (*p*=0.002, *p*=0.009). The mean levels of triglycerides and glucose were outstandingly higher in MS+ group in comparison with MS- group (*p*<0.001 for both).

While before the bariatric surgery 24 patients met criteria corresponding to metabolic syndrome, after the surgery the number fell to 13 patients only. In comparison with MS- patients, the MS+ patients had more prominent changes in some blood parameters after surgery, including decrease of cholesterol, LDL-cholesterol and triglycerides, and increase of vitamin B9 (Table 2).

**Table 2:** Percent of changes from baseline (before bariatric surgery) to 12 months after surgery in clinical characteristics, levels of adipokines and metabolic indices in patients with and without metabolic syndrome

Indices	Change in 12 months MS+ patients mean % change (95% CI)	Change in 12 months MS- patients mean % change (95% CI)	*P*-value for MS+ patients	*P*-value for MS- patients
BMI (kg/m^2^)	-30.4 (-23.4 to -37.3)	-46.3 (-61.6 to -30.9)	***p*<0.001**	***p*<0.001**
hs-CRP (mg/ml)	-62.5 (-42.7 to -82.2)	-300 (-592 to -7,9)	***p*<0.001**	***p*<0.001**
Cholesterol (mmol/l)	-12.5 (-18.2 to -6.7)	3.8 (-2.2 to 9.8)	***P*=0.031**	NS
LDL-Cholesterol (mmol/l)	-14.1 (-41.6 to 13.4)	-9.8 (-22.8 to 3.2)	***P*=0.009**	NS
HDL-Cholesterol (mmol/l)	32.8 (10.8 to 54.7)	32.7 (12.2 to 53.1)	***P*=0.007**	***P*=0.026**
Triglycerides (mmol/l)	-37.2 (-26.2 to -48.2)	-16.5 (-53.9 to 20.9)	***P*=0.005**	NS
Glucose (mmol/l)	-23.0 (-31.7 to -14.2 )	-13.7 (-22.7 to -4.6)	***P*=0.040**	***P*=0.049**
A1C (%)	-20.1 (-11.38 to -28.8)	-9.2 (-18.3 to -0.07)	***P*=0.003**	***p*=0.038**
A1C (IFCC) (mmol/l)	-13.5 (-6.8 to -20.1)	-5.0 (-10.5 to -0.4)	***P*=0.003**	***P*=0.041**
Vitamin D (nmol/l)	8.53 (-9.5 to 26.5)	40.3 (9.2-71.3)	NS	***P*=0.05**
Adiponectin (μg/ml)	118.0 (81.1-154.8)	81.4 (55.0 to 107.7)	***P*<0.001**	***P*=0.032**
Resistin (ng/ml)	15.3 (-5.3 to 35.9)	6.3 (-21.7 to 34.4)	NS	NS
Leptin (ng/ml)	-65.4 (-84.2 to -46.5)	-78.4 (-90.3 to -66.4)	***P*<0.001**	***P*<0.001**
Vitamin B12 (pmol/l)	41.92 (-27.3 to 111.2)	-14.3 (-32.9 to 4.3)	NS	NS
Vitamin B9 (nmol/l)	63.8 (17.6-109.9)	41.8 (-24.1 to 107.7)	***P*=0.022**	NS
Ferritin (μg/l)	-16.1 (-56.6 to 24.4)	-23.0 (-55.6 to 9.6)	NS	NS
Fe (μmol/l)	33.6 (-18.05 to 85.2)	43.2 (-9.6 to 96.8)	NS	NS

*BMI* body mass index, *MS* metabolic syndrome, *hsCRP* C-reactive protein, *A1C* glycated hemoglobinm, *LDL-cholesterol* low density cholesterol, *HDL-cholesterol* high density cholesterol, *ND* not detected, *NS* not significant, *Fe* iron

**Fig. 4:**
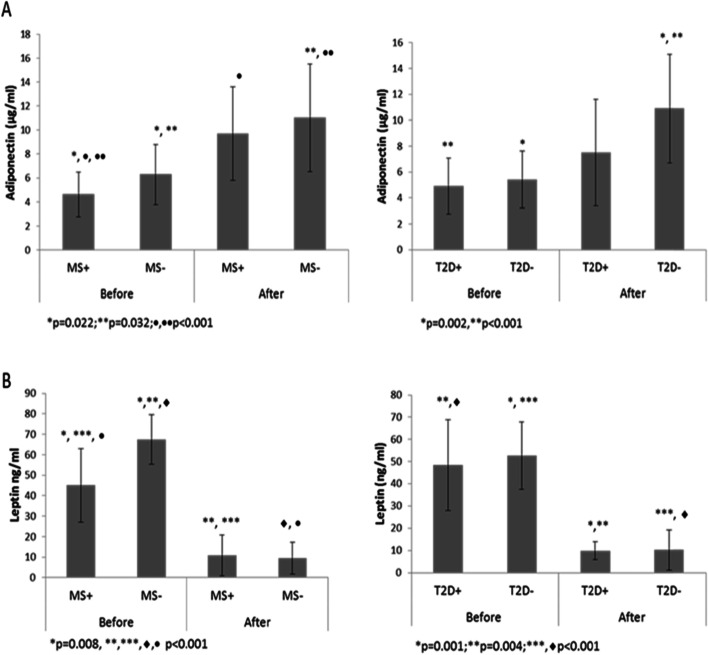
Baseline plasma levels of adiponectin (**A**) and leptin (**B**) among patients with/without metabolic syndrome and type 2 diabetes before and after bariatric surgery. Values represent mean±SD plasma levels. The x-axis represents the groupings of patients according to study groups, the y-axis represents plasma concentration of relative adipokines

For bariatric surgery patients the levels of both adiponectin and leptin before surgery sharply differentiated between MS+ and MS- patients. After bariatric surgery the level of adiponectin similarly increased in both groups, while the decrease of leptin level after bariatric surgery was higher in MS-group (*p*<0.001**,** Fig. 4).

### Comparison of obese patients with and without type 2 diabetes

No significant differences in serum levels of adipokines between the obese patients with and without T2D were found (Supplementary Table S4). Before surgery, hs-CRP levels were higher in T2D+ group in comparison with T2D- group (*p*>0.001). After bariatric surgery, the level of hs-CRP decreased to the similar value in both study groups. Post-surgery T2D- group had lower BMI in comparison with post-surgery T2D+ group (*p*>0.001).

After bariatric surgery, the number of T2D+ patients decreased by 86.3% (from 22% to 3%). Compared with T2D+ group, the T2D-patients showed significantly higher levels of adiponectin after bariatric surgery. Reduction of leptin was similar in both subgroups (Fig. 4).

## Discussion

Our study revealed significant changes in the levels of some adipokines among obese patients after bariatric surgery: the levels of adiponectin increased while those of leptin decreased 1 year after surgery. In addition, positive changes in metabolic biomarkers were noted after bariatric surgery, including the blood levels of glucose, glycated hemoglobin, hsCRP, cholesterol and its fractions, triglycerides as well as vitamins D and B9. At the same time, though the levels of resistin were substantially lower in the control group than in the obese patients, their decrease after bariatric surgery was insignificant.

We recruited three different groups into our study: healthy controls with normal BMI, obese patients from GP practice and obese patients going to bariatric surgery. The latter were investigated before surgery and 1 year after surgery when they experienced remarkable weight loss (>40 kg). Similar weight loss has also been described in previous studies [23–26]. We found that the mean levels of triglycerides, glucose and glycated hemoglobin in the patients qualified for surgery were higher than in the control group, that is also in agreement with previous studies [27, 28]. We also observed several beneficial tendencies in blood markers following a 12-month follow-up period like decrease of glucose, glycohemoglobin, hsCRP, cholesterol, LDL-cholesterol and triglycerides levels, and increase of HDL-cholesterol, levels of vitamins D and B9. Similar tendencies were revealed in previous studies, too [29, 30]. These changes confirm beneficial impact of bariatric surgery on the metabolic processes, in addition to weight loss.

A cluster of risk factors for cardiovascular disease and type 2 diabetes have become known as the metabolic syndrome. These risk factors include some above-mentioned blood markers like dyslipidemia (raised triglycerides and HDL-cholesterol) and raised fasting glucose, but also raised blood pressure and central obesity [22]. Thus, in patients with visceral obesity, an adipocytokine imbalance develops, contributing to the development of several unwanted conditions like insulin resistance, type 2 diabetes, hyperlipidemia, stroke, atherosclerosis [10–12]. In obesity, the synthesis and secretion of proinflammatory adipokines are up-regulated whereas the production of the major anti-inflammatory adipokine is down-regulated. Twenty four of the 30 pre-surgery obese patients in our study had metabolic syndrome that resolved in eleven cases after surgery.

In our study, the lowest levels of adiponectin were detected in BS group before surgery. It almost doubled after bariatric surgery and stayed even higher than in the control group although some operated patients were still slightly overweight. Besides, the moderate positive correlation between the level of adiponectin and patient’s age was marked. Kirillova et al. demonstrated a negative correlation between adiponectin level and BMI in morbidly obese patients, which is congruent with our data [31]. Adiponectin is important in transporting glucose, regulating lipid metabolism and improving insulin sensitivity, which elicit an anti-inflammatory role in inhibiting the formation of atherosclerosis [28, 32, 33]. The revealed inverse correlation between adiponectin concentration and BMI in obese patients allows us to conclude that adipose tissue with increasing fat mass decreases fatty acid oxidation in muscles and liver, and this, apparently, contributes to lipid accumulation in the cells.

Regarding leptin, the highest levels were seen in obese patients being moderately correlated with weight and BMI in females. Bariatric surgery had positive effect similarly to adiponectin, and leptin levels decreased substantially in both males and females. It was somewhat surprising that the mean levels of leptin differed between two groups of obese patients, and this difference was more pronounced in women. At the same time, the norm values are also significantly different in men and women, and most of the obese patients also fitted into the norm values before surgery. The amount of leptin circulating in the body is proportional to the volume of fatty tissue in human body [34]. In women, leptin may act as a critical link between adipose tissue and hypothalamic centers for regulation of energy homeostasis [35]. Our finding is in accordance with other studies demonstrating that serum leptin levels drop in response to weight loss [35, 36]. Furthermore, changes in serum leptin level positively correlated with changes in BMI in women who underwent laparoscopic gastric banding [37].

In our study, the lowest level of resistin was observed in the control group. At the same time, there was no statistically significant change after bariatric surgery though decreasing tendency was observed. Similarly, no significant differences in resistin levels between morbidly obese patients and healthy subjects of normal weight, or between obese patients before and after weight loss was found earlier [38]. At the same time, quite strong correlation between level of resistin, weight and BMI, and moderate correlations for glucose and glycated hemoglobin were found in our study.

An important recent evidence connects obesity, diabetes and a state of chronic low-grade inflammation [39]. Resistin may play a causal role in the development of insulin resistance and T2D, however this role remains still controversial [40–44]. In humans, resistin lies on chromosome 19p13.3, a region that has not been linked with susceptibility to obesity [45]. Similarly to our study, rather weak correlation was established between plasma glucose concentration and resistin level in morbidly obese patients in a study of Kirillova et al. [31]. At the same time, Al-Harithy and Al-Ghamdy showed significant positive resistin correlation with BMI and plasma glucose in diabetic women, while for non-diabetic women and lean persons no such correlation was found [46]. Some studies found that plasma resistin levels were significantly decreased 12 months after bariatric surgery with >10% loss of the excess body weight ( [47–49]. Thus, conflicting results exist as concerns resistin.

With regard to surgery method, as our results show, the LRYGB method was more effective in reducing the level of resistin than LSG method. The significantly increased level of resistin was demonstrated for restrictive bariatric surgery in premenopausal morbidly obese women [35]. Effects of other bariatric surgery methods on plasma resistin levels are inconclusive. We didn’t find any differences between two surgical methods in case of other adipokines or metabolic blood parameters. Previously, an improvement in metabolic syndrome parameters was reported for all surgery techniques, but in case of LRYGB the improvement was even greater [50].

We also compared the obese patients with and without MS. Some variations in changes of metabolic blood markers and adipokines in these patients following a lapse of 12 months after bariatric surgery were observed. A significant improvement in adiponectin and some metabolic parameters (cholesterol, LDL-cholesterol, triglycerides) was noted for MS+ patients. Similarly, Yadav et al. demonstrated that patients with diabetes led to a dramatically greater reduction in triglycerides [30]. Bariatric surgery may induce considerable and persistent improvement in prevalence of MS through decrease of amount of excess weight lost [51].

Certain changes in vitamin levels after surgery were noted. The levels of vitamin D in blood were dramatically low in majority of study groups. Only in 10-11% of obese patients and in 20% of control group patients the vitamin D levels were in normal range. This phenomenon is most likely related to the deficiency of sun in a Nordic country [52]. Previously it has been demonstrated that gastric banding may increase the risk for metabolic bone disease due to the inadequate intake of calcium and vitamin D in the immediate postoperative period [53, 54]. The bariatric surgery patients are advised to always use vitamin and mineral supplements such us as calcium and iron [55]. In Estonia, the postoperative patients were advised to regularly take multivitamins containing at least 200 μg of folic acid (100% Recommended Dietary Allowance [RDA]), 14 mg of iron (100% RDA), 1 μg of vitamin B12 (40% RDA), zink and selenium [24]. Unfortunately, we do not have accurate data about the patients’ habits to consume these food supplements and vitamins. However, there is a study of 5-year follow-up demonstrating that 50% of the patients consumed vitamins regularly and 14% took them irregularly [24]. In our study, only in 37% of patients the vitamin D level increased higher than normal 12 months after bariatric surgery. Though the mean levels were increased after surgery, they still remained below the reference value in remarkable proportion of the patients. The low vitamin D status may link to the dysregulation of white adipose tissue [56]. In the present study the negative relationship between low levels of vitamin D and resistin was also found. However, previously similar relationship in patients with morbid obesity was not marked [57].

Additionally, we demonstrated significant increase of folic acid (vitamin B9) blood level in patients with metabolic syndrome after surgery. This vitamin helps to control metabolism, as well as to break down carbohydrates, proteins and fats into energy [58]. The changes in level of vitamin B9 may be associated with changed intestinal microbiota, since some intestinal bacteria like *Lactobacillus* sp., *Bifidobacterium* sp. can produce folic acid [59]. As for vitamin B12, usually deficiency of this vitamin develops within 1-4 years after bariatric surgery because of the lack of intrinsic factor (IF) which is synthesized in gastric parenteral cells and in the case of its shortage, the vitamin B12 cannot absorb [60–62].

Limitations of our study include moderate size of study groups lacking exact age and gender match. Moreover, longer follow-up of the operated patients would provide more important information about the long-term consequences.

## Conclusions

The remarkable changes in the levels of adipokines after bariatric surgery appear like increase in adiponectin and decrease in leptin levels. Additionally, significant improvement occurs in anthropometric parameters, metabolic and inflammatory markers, suggesting high potential for reduction of metabolic syndrome and risk of type 2 diabetes. We have shown for the first time ever that level of vitamin D may be involved in resistin regulation. Further detailed studies with increased number of patients and longer follow-up observation time are needed to conclusively address this topic.

## Supplementary Information

**Additional file 1: Supplementary table 1.** The values of anthropometric and metabolic variables in male and female subjects. **Supplementary table 2.** The values of adipokines in study subjects. **Supplementary table 3.** The values of anthropometric and blood variables in all patients with (MS+) and without (MS-) metabolic syndrome. **Supplementary table 4.** The values of anthropometric and blood variables measured in the subjects with (T2D+) and without (T2D-) type 2 diabetes.

## Data Availability

The datasets used and analysed during current study available from the corresponding author on reasonable request.

## Abbreviations

LSG: Laparoscopic sleeve gastrectomy
LRYGB: Laparoscopic gastric bypass
MS: Metabolic syndrome
T2D: Type 2 diabetes
hsCRP: C-reactive protein
A1C: Glycated hemoglobin
BMI: Body mass index
