# Supplementary material for: Changes in adipokine levels and metabolic profiles following bariatric surgery
Source: BMC Endocr Disord. 2022 Feb 3;22:33. doi: 10.1186/s12902-022-00942-7 (PMC8812034; doi:10.1186/s12902-022-00942-7)
Supplement: Supplementary file 1 — Additional file 1: Supplementary table 1. The values of anthropometric and metabolic variables in male and female subjects. Supplementary table 2. The values of adipokines in study subjects. Supplementary table 3. The values of anthropometric and blood variables in all patients with (MS+) and without (MS-) metabolic syndrome. Supplementary table 4. The values of anthropometric and blood variables measured in the subjects with (T2D+) and without (T2D-) type 2 diabetes. [file 12902_2022_942_MOESM1_ESM.doc]

**Supplementary table 1**. The values of anthropometric and metabolic variables in male and female subjects

| **Indices** | | **Reference values**  **mean±SD (range)** | **Mean±SD [median ((25th and 75th percentile)]** | | | | | | | | ****P-value** |
| --- | --- | --- | --- | --- | --- | --- | --- | --- | --- | --- | --- |
|  |  |  | **Obese patients in GP practice** | | **Obese patients going to bariatric surgery** | | | | **Control group** | |  |
|  |  |  |  |  | **Before surgery** | | **1 year after surgery** | |  |  |  |
|  |  |  | Male  n=40 | Female  n=20 | Male  n=11 | Female  n=19 | Male  n=11 | Female  n=19 | Male  n=6 | Female  n=9 |  |
| Age (years) | |  | 51.8±10.5 [53(43.3-58.8)]^e^ | 49.3±9.6 [48(41.5-58)]^f^ | 45.5±6.5 [47(41-52)]^a^ | 47.7±10.1 [50(40-56)]^b^ | 46.7±6.5 [48(42-53)]^c^ | 49±10.1 [52(42-57)]^d^ | 30±9.7 [28(22.5-37.3)]^a,c,e^ | 39.7± 6.9 [40(36-42)]^b,d,f^ | ^a^ p=0.009  ^b^ p=0.04  ^c,f^ p=0.006  ^d^ p=0.028  ^e^ p=0.002 |
| Weight (kg) | |  | 101.6±14.0 [99.6(90.8-110.0)]^c,g,m^ | 114.1±11.7 [116.3(104.5-122.8)]^c,n,o^ | 147.0±24.1 [151(132-158)]^a,e,g,h^ | 118.7±14.7[118(106.0-125.0)]^a,f,i^ | 99.7±21.1 [93(86-113)]^b,e,k^ | 78.7±12.5 [77.0(70-9) ]^b, f, j, l^ | 76.7±11.1 [75.5(66.8-87.5)]^d,h,k,m^ | 65.7±8.0 [(59.0-71.5)]^d,i,j,l,n,o^ | ^a^ p=0.003  ^b*^ p=0.002  ^c*,e, f*^, ^g*,h, i, j, m, n,o^ p<0.001  ^d^ p=0.042  ^k^ p=0.01  ^l^ p=0.005 |
| Weight lost (kg) | |  | - | | - | | 47.3±14.0(31.0-70.0) [42(34-59)] | 40.1±8.5 (23.0-55.0) [37.0(35.0-48.0)] | - | | NS |
| BMI (kg/m^2^) | |  | 36.9±4.6 [36.4(33.7-39.9)]^a,d,h,l,m*^ | 34.0±3.3 [34.1(31.8-36.9)]^a,e,i^ | 44.5±5.3[44.0(40.0-50.0)]^b,d,f,h^ | 44.4±5.8 [44.0(40.0-46.0)]^c,e,g,i^ | 29.9±5.9 [28.0(26.0-35.0)]^b,j,m^ | 29.3±3.9 [30.0(26.0-32.0)]^c,k^ | 22.2±2.6 [22.0(19.8-24.5)]^f,j,l^ | 23.3±1.9 [24.0(22.0-24.5)]^g,k^ | ^a*^ p= 0.017  ^b,c,d*,e ,f, g*,i*,j,k*,l, m*^ p<0.001  ^h^ p=0.001 |
| Adiponectin (µg/ml) | | 6.6 (0.9-21.4) | 8.1±2.7 [8.3(5.8-10.8)]^b,e^ | 4.6±4.1 [3.7(2.5-4.7)]^b,f,h,j^ | 3.4±1.6  [33.0(91.9-4.2)]^a,c,e,g^ | 6.1±1.8  [6.0(5.1-7.1)]^a,d,f^ | 8.9±4.8 [8.6(4.1-14.1)]^c^ | 11.6±3.7 [11.7(8.7-15.4)]^d,h,i^ | 6.9 ±2.6 [6.5(4.9-8.8)]^g^ | 7.7±4.1  [5.5(5.1-9.6)]^i,j^ | ^a,b,d,e,h,j^  p<0.001  ^c^ p=0.003  ^f^ p=0.001  ^g,i^ p=0.002 |
| Leptin (ng/ml) | Male | 4.8  (2.2-11.2) | 46.1±21.9 [43.7(27.652.0)]^c,f,k,l^ | 14.0±8.9  [10.7(7.7- 18.2)]^c,h^ | 31.3±10.5 [26.9(22.340.5)]^a,e,f,i^ | 60.2±14.3 [61.1(48.5-66.9)]^a,g,h,j^ | 6.1±5.5 [3.6(2.0-14.0)]^b,e,k^ | 12.5±9.4 [8.2(7.1-15.7)]^b,g^ | 2.8±1.7 [2.3(1.4-4.4)]^d,i,l^ | 13.0±7.5 [11.6(6.6-19.3)]^d, j^ | ^a,c,e,f,h,i,j,k,l^ p<0.001  ^b,g^ p=0.02  ^d^ p=0.006 |
|  | Female | 20.7  (3.9-77.3) |  |  |  |  |  |  |  |  |  |
| Resistin (ng/ml) | | 13.8 ±4.6 (6.4-26.4) | 14.3±8.0 [13.2(8.6-16.9)] | 13.3±7.5 [10.6(7.8-17.3)] | 15.7±7.1 [16.3(9.3-20.0)] | 16.7±8.6 [14.4(9.3-21.1)]^a^ | 14.3±7.9 [10.6(8.8-22.0)] | 13.8±6.2 [12.0(9.5-18.7)] | 11.5±4.8 [12.4(7.1-15.8)] | 9.6±3.2 [8.8(7.3-11.7)]^a^ | ^a^ p=0.019 |
| hs-CRP (mg/L) | | <5.0 | 4.7±4.9 [2.8(1.2-7.4)]^f^ | 3.0±2.8 [2.2(1.0-4.0)]^c^ | 7.4±6.2 5[(4-12)]^a, d^ | 5.2±5.0 [3(2-6)]^b, c, e^ | 1.1±0.3 [1(1-1)]^a, f^ | 1.3±0.67 [1(1-1)]^b^ | 1.8±1.0 [1.5(1.0-2.8)]^d^ | 1.0±0.0 [1(1-1)]^e^ | ^a,b,d*^ p<0.001  ^c*^ p=0.03  ^e^ p=0.002  ^f*^ p=0.02 |
| Cholesterol (mmol/l) | | <5.0 | 5.5±1.1 [5.6(4.7-6.3)]^b^ | 5.7±1.0 [5.6(5.2-6.3)]^c^ | 5.1±0.4 [5.2(4.7-5.4)]^a^ | 5.2±1.2 [4.8(4.3-6.3)] | 4.4±0.4 [4.3(4.1-4.8)]^a,b^ | 4.6±0.8 [4.6(4.0-5.3)]^c^ | 5.0±0.8 [5.2(4.2-5.6)] | 5.2±1.2 [5.1(4.1-5.9)] | ^a,b,c^ p<0.001 |
| LDL–cholesterol (mmol/l) | | <3.0 | 3.5±1.0 [3.4(2.8-4.2)]^c^ | 3.7±0.9 [3.6(3.2-4.3)]^d^ | 3.4±0.5 [3.1(3.0-4.0)]^a^ | 3.4±1.2 [3.1(2.6-4.5)]^b^ | 2.6±0.4 [2.7(2.2-3.0)]^a,c,e^ | 2.7±0.6 [2.8(2.1-3.1)]^b,d,f^ | 3.7±0.7 [3.9(3.0-4.2)]^e^ | 3.6±0.8 [3.4(2.9-4.3)]^f^ | ^a, c, d^  p<0.001  ^b^ p=0.02  ^e^ p=0.001  ^f^ p=0.006 |
| HDL–cholesterol  (mmol/l) | | >1.0 | 1.4±0.3 [1.4(1.2-1.7)]^d, e^ | 1.3±0.3 [1.3(1.1-1.5)]^f, g^ | 1.1±0.3 [1.1(0.9-1.2)]^a, b, d^ | 1.6±0.8 [1.3(1.2-1.6)]^a, c^ | 1.7±0.5 [1.6(1.5-2.1)]^b, e^ | 1.8±0.4 [1.8(1.6-2.3)]^c, f^ | 1.2±0.3 [1.2(0.9-1.6)] | 1.8±0.5 [1.7(1.3-2.0)]^g^ | ^a^ p=0.008  ^b,c,d,e,g^ p=0.002  ^f^ p<0.001 |
| Triglycerides (mmol/l) | | <1.7 | 1.7±1.0 [1.4(1.1-1.8)]^c^ | 2.1±1.3 [1.5(1.1-3.0)]^e,f^ | 2.2±1.1  [2.0(1.6-2.3)]^a,c^ | 1.6±0.5 [1.5(1.1-2.0)]^b,d^ | 1.2±0.6 [1.2(0.6-2.0)]^a^ | 1.1±0.4 [0.9(0.8-1.5)]^b,e^ | 1.7±1.1 [1.3(0.9-2.8)] | 0.9±0.2  [0.9(0.8-1.0)]^d,f^ | ^a^* p=0.008  ^b,c^* p=0.003  ^e^* p=0.002  ^d,f^* p=0.005 |
| Glucose (mmol/l) | | 4.1-6.1 | 5.8±0.9 [5.6(5.1-6.1)]^e^ | 5.8±0.9 [5.8(5.2-6.1)]^d,f^ | 8.0±3.4 [6.9(5.1-9.7)] | 6.0±1.2 [5.8(5.4-6.5)]^a,b,c^ | 5.8±1.4 [5.4(4.9-6.4)]^e^ | 4.8±0.8 [4.8(4.6-5.1)]^a,b,d^ | 4.7±0.8  [4.8(3.9-5.5)] | 4.9±0.6 [5.0(4.4-5.4)]^c,f^ | ^a,e*,f^ p=0.03  ^b,d*^ p<0.001  ^c*^ p=0.01 |
| A1C (%) | | 4.0-6.0 | 5.7±0.4 [5.6(5.4-5.7)]^e,g^ | 5.6±0.4 [5.6(5.3-5.8)]^f, h^ | 6.6±1.9 [5.7(5.5-8.0)]^a,c^ | 5.7±0.3 [5.7(5.5-5.9)]^b,d^ | 5.4±0.6 [5.2(5.0-5.7)]^a,e^ | 5.3±0.3 [5.3(5.1-5.4)]^b,f^ | 5.1±0.2 [5.1(4.9-5.3)]^c,g^ | 4.±0.31 [5.1(4.6-5.2)]^d,h^ | ^a,d^ p=0.03  ^b^ p<0.001  ^c, e*^ p=0.02  ^f^ p=0.007  ^g*^ p=0.003  ^h^ p=0.005 |
| HbA1c (IFCC) (mmol/l) | | 20.0-42.0 | 38.3±3.9 [37.7(35.5-39.0)]^e,g^ | 37.6±4.4 [37.7(34.7-39.9)]^f,h^ | 48.5±20.2 [40.0(37.0-60.0)]^a,c^ | 38.7±3.2 [39.0(37.0-41.0)]^b,d^ | 35.3±6.1 [33.0(31.0-39.0)]^a,e^ | 34.1±3.6 [33.5(32.0-36.3)]^b,f^ | 32.0±1.8 [32.0(30.3-33.8)]^c,g^ | 30.4±3.1 [32.0(27.0-33.0)]^d,h^ | ^a *^p=0.02  ^b,d^ p<0.001  ^c*^ p=0.01  ^f^ p=0.007  ^g*^ p=0.003  ^h^ p=0.004 |
| Vitamin D (25-OH) (nmol/l) | | >75.0 | 53.5±16.3 [50.9(42.8-62.9)]^a,d^ | 39.5±14.4 [38.6(29.25-46.5)]^a,c,e,f^ | 55.8±16.6 [47.5(40.7-65.8)] | 53.3±14.0 [54.8(40.1-62.6)]^b,c,d^ | 68.4±18.7 [72.1(47.4-77.4)]^d^ | 67.6±23.6 [68.7(49.0-81.7)]^b,e^ | 45.1±23.2 [40.9(25.8-68.6)]^d^ | 111.2±92.6[78.4(57.0-181.8)]^f^ | ^a^ p=0.02  ^b,d,f*^ p=0.03  ^c^ p=0.005  ^d^ p=0.01  ^e^ p<0.001 |
| Vitamin B12 (pmol/l) | | 145.0-569.0 | ND | ND | 305.8±105.0  [291.0(226.0-360.0)] | 303.6±112.6  [298.0(237.0-342.0)] | 315.1±109.2  [321.0(219.0-395.0)] | 287.2±126.7  [260.5(198.8-322.3)] | 404.8±161.4  [437.0(237.5-539.8)] | 284.2±92.2  [250.0(209.5-376.0)] | NS |
| Vitamin B9  (nmol/l) | | 8.8-60.8 | ND | ND | 15.1±6.4  [16.6(8.4-20.8)]^a^ | 15.5±8.2  [12.5(10.9-20.3)]^b,c^ | 24.2±11.3  [23.9(12.3-35.4)]^a,b^ | 31.8±28.6  [16.3(9.6-61.5)]^c^ | 17.7±9.7  [14.4(10.7-28.0)] | 13.0±1.7  [13.1(11.5-14.6)] | ^a^p=0.03  ^b,c^p=0.02 |
| Ferritin (µg/l) | Male | 30.0–400.0 | ND | ND | 202.3±82.9  [245.1 (127.0-262.0)]^a,b,c^ | 110.3±66.6  [84.0(60.8-154.2)]^a,d,e^ | 192.8±89.5  [176.8(116.8-247.1)]^d,f^ | 114.1±89.7  [78.6(34.8-203.2)]^b,f,g^ | 226.3±86.7  [203.7(157.7-317.7)]^e,g^ | 87.1±90.7  [47.9(40.2-153.6)]^c^ | ^a^p=0.002  ^b^p=0.01  ^c,f,g^p=0.03  ^d^p=0.008  ^e^p=0.007 |
|  | Female | 13.0–150.0 |  |  |  |  |  |  |  |  |  |
| Fe  (µmol/l) | Male | 10.6-28.3 | ND | ND | 19.1±5.7  [17.8 (13.9-25.1)] | 16.2±6.0  [15.0(12.6-18.7)]^a^ | 22.3±6.6  [20.2(17.3-25.1)]^a^ | 20.6±9.3  [19.2(15.1-24.1)] | 18.9±3.2  [19.2(15.8-21.7)] | 17.7±3.8  [18.5(14.9-20.2)] | ^a^p=0.02 |
|  | Female | 6.6-26.0 |  |  |  |  |  |  |  |  |  |

* Mann Whitney Rank Sum Test; **t-test; NS-not significant, ND-not detected, A1C – glycated hemoglobin; Fe-iron.

**Supplementary table 2.** The values of adipokines in study subjects

| Indices | | Reference values  mean±SD  (range) | Mean±SD [median (25th and 75th percentile)] | | | | P-value |
| --- | --- | --- | --- | --- | --- | --- | --- |
|  |  |  | Obese patients in GP practice (n=60) | Obese patients going to bariatric surgery (n=30) | | Control group (n=15) |  |
|  |  |  |  | Before surgery | 1 year after surgery |  |  |
| Adiponectin (µg/ml) | | 6.6 ±3.7  (0.8-21.4) | 6.9±3.6  [6.6(3.8-9.2)]^b,d^ | 5.1±2.2  [5.2(3.5-6.5)]^a,b,c^ | 10.6±4.3  [9.9(7.5-14.2)]^a,d,e^ | 7.4±3.5  [5.5 (5.2-8.7)]^c,e^ | ^a,d^ p<0.001  ^b*,c*^ p=0.03  ^e p^=0.01 |
| Leptin (ng/ml) | Male | 4.8  (2.2-11.2) | 46.1±21.9 [43.8(27.7-52.0)]^b,d,e^ | 31.3±10.5 [26.9(22.3-40.5)]^a,b,c^ | 6.1±5.5 [3.6(2.0-14.0)]^a,d^ | 2.8±1.7 [2.3(1.4-4.4)]^c,e^ | ^a,c,d*,e*^ p<0.001  ^b, p^=0.02 |
|  | Female | 20.7  (3.9-77.3) | 14.0±8.9  [10.7(7.7-18.2)]^b^ | 60.2±14.3  [61.1(48.5-67.0)]^a,b,c^ | 12.5±9.4 [8.2(7.1-15.7)]^a^ | 13.0±7.5  [11.6(6.6-19.3)]^c^ | ^a*,b,c*^ p<0.001 |
| Resistin (ng/ml) | | 13.8 ±4.6  (6.4-26.4) | 14.0±7.8  [12.0(8.6-17.0)]^c^ | 16.3±8.0  [14.5(9.3-20.7)]^a^ | 14.0±6.7  [11.2(9.5-20.1)]^b^ | 10.4±3.9  [10.4(7.8-13.3)]^a,b,c^ | ^a*^p=0.009  ^b^ p=0.006  ^c^ p<0.001 |

*Mann Whitney Sum Rank Test.

**Supplementary table 3**. The values of anthropometric and blood variables in all patients with (MS+) and without (MS-) metabolic syndrome

| Indices | | Mean±SD [median ((25th and 75th percentile)] | | | p-value |
| --- | --- | --- | --- | --- | --- |
|  |  | Obese patients with metabolic syndrome (n=58; 32 M and 26 F) | Obese patients without metabolic syndrome (n=25; 16 M and 9 F) | Control group  (n=15; 6 M and 9 F) |  |
| Age (years) | | 50.3±9.6  [50.5(43-58)]^a^ | 47.4±10.2  [48(39-54)]^b^ | 35.8±9.2  [37(28-4)]^a,b^ | ^a,b^ p<0.001 |
| Weight (kg) | | 118.5±44.3  [113.5(104.4-128.1)]^a,b^ | 103.3±13.9  [100.0(91.0-115.5)]^a,c^ | 70.1±10.  [69.0(63.0-76.0)]^b,c^ | ^a^ p=0.002  ^b,c^ p<0.001 |
| BMI (kg/m2) | | 40.2±6.6  [40.0(34.9-44.0)]^a,b^ | 36.2±5.1  [36.4(31.6-40.2)]^a,c^ | 22.9±2.2  [23.0(21.0-24.0)]^b,c^ | ^a^ p=0.009  ^b,c^ p<0.001 |
| Adiponectin (µg/ml) | | 6.0±3.5  [5.6(3.7-7.2)] | 6.8±3.2  [6.6(4.4-9.3)] | 7.4±3.5  [5.5(5.2-8.7)] | NS |
| Leptin (ng/ml) | Male | 46.5±23.2  [42.2(26.9-54.9)]^a^ | 38.0±15.1  [38.5(23.8-48.3)]^b^ | 2.8±1.7  [2.3(1.4-4.4)]^a,b^ | ^a,b^ p<0.001 |
|  | Female | 34.9±24.8  [33.4(11.0-59.1)]^a^ | 30.1±26.1  [24.5(6.0-59.0)] | 13.0±7.5  [11.6 (6.6-19.3)]^a^ | ^a^ p=0.01 |
| Resistin (ng/ml) | | 15.2±7.4  [14.2 (9.4-20.1)]^a^ | 14.9±9.5  [14.2 (7.7-18.2)] | 10.4±3.9  [10.4 (7.8-13.3)]^a^ | ^a^ p=0.02 |
| hs-CRP (mg/l) | | 5.0±5.03  [3.0(1.4-6.8)]^a^ | 3.4±4.0  [2.0(1.0-4.4)] | 1.3±0.71  [1.0(1.0-1.5)]^a^ | ^a^ p=0.03 |
| Cholesterol (mmol/l) | | 5.5±1.1  [5.4(4.6-6.1)] | 5.4±1.0  [5.5 (4.8-6.2)] | 5.1±1.0  [5.2(4.1-5.2)] | NS |
| LDL–cholesterol (mmol/l) | | 3.5±1.0  [3.3(2.8-4.2)] | 3.5±1.0  [3.6(3.1-4.3)] | 3.6±0.7  [3.8(2.9-4.1)] | NS |
| HDL–cholesterol (mmol/l) | | 1.4±0.6  [1.2(1.0-1.6)] | 1.4±0.3  [1.4 (1.3-1.7)] | 1.6±0.5  [1.5(1.2-1.8)] | NS |
| Triglycerides (mmol/l) | | 2.1±1.1  [1.9(1.3-2.5)]^a,b^ | 1.3±0.5  [1.2(1.0-1.5)]^a^ | 1.2±0.7  [0.9 (0.2-1.2)]^b^ | ^a^ p<0.001  ^b^ p=0.02 |
| Glucose (mmol/l) | | 6.6±1.8  [6.0 (5.6-6.0)]^a,b^ | 5.2±0.6  [5.1(4.-5.34)]^a^ | 4.81±0.67  [5.00(4.30-5.40)]b | ^a^ p<0.001  ^b^ p=0.009 |
| A1C (%) | | 5.9±0.9  [5.6(5.4-6.0)]^a^ | 5.7±0.3  [5.6(5.3-5.7)]^b^ | 5.0±0.3  [5.1(4.8-5.2)]^a,b^ | ^a,b^ p<0.001 |
| A1C (IFCC) (mmol/l) | | 40.57±10.03  [38.4(35.5-42.03)]^a^ | 37.52±3.44  [37.7(35.00-39.00)]^b^ | 31.1±2.6  [32.0(28.5-33.0)]^a,b^ | ^a^ p=0.02  ^b^ p<0.001 |
| Vitamin D (25-OH) (nmol/l) | | 50.7±17.1  [49.6(38.9-61.7)]^a^ | 51.9±15.9  [48.8 (40.6-63.4)]^b^ | 81.8±75.5  [60.2(40.0-83.5)]^a,b^ | ^a^ p<0.02  ^b^ p=0.05 |

The levels of vitamins B12, B9, Ferritin and Fe are not present in this table such as the amounts of MS+ and MS- patients with these parameters were limited. A1C – glycated hemoglobin; Fe-iron.

NS - not significant.

**Supplementary table 4**. The values of anthropometric and blood variables measured in the subjects with (T2D+) and without (T2D-) type 2 diabetes

| **Mean±SD [median (25th and 75th percentile )]** | | | | | | | | ****p-value** |
| --- | --- | --- | --- | --- | --- | --- | --- | --- |
| **Indices** | | | **Reference values**  **mean±SD**  **(range)** | **T2D+ (n=22)** | | **T2D- (n=8)** | |  |
|  |  |  |  | **Before surgery** | **1 year after surgery** | **Before surgery** | **1 year after surgery** |  |
| Age (years) | | |  | 47±8.2  [47.5(40.8-53)] | 48.2±8.3 [48.5(42.8-54)] | 46.8±11.1 [51(38-55.5)] | 48.0±11.0  [52.5(39.8-56.5] | NS |
| Weight (kg) | | |  | 133.1±24.6 [126.5(113.8-154.5)]^a,b^ | 89.9±20.3 [85.5(77.0-97.3)]^a,c^ | 118.1±13.5 119.0[(105.3-129.5)]^c,d^ | 76.9±10.4 [76.0(67.5-85.0)]^b,d^ | ^a,b,d^ p<0.001  ^c^ p=0.001 |
| BMI (kg/m^2^) | | |  | 45.1±6.2  [44.0(40.0-49.3)]^a,b^ | 30.3±5.0 [30.0(26.8-33.0)]^a,c,d^ | 42.6±3.0 [42.5(40.0-45.5)]^e^ | 27.4±2.0  [26.5(25.3-29.5)]^b,c,d,e^ | ^a*,b*,c,d,e^  p<0.001 |
| Adiponectin (µg/ml) | | | 6.6±3.7  (0.9-21.4) | 4.9±2.2  [5.3(3.4-6.5)]^a,b^ | 10.3±4.3  [9.6(7.3-14.2)]^a,c^ | 5.4±2.3 [5.2(3.6-6.6)]^c,d^ | 11.5±4.1  [11.1(8.6-14.8)]^b,d^ | ^a,c^ p<0.001  ^b^ p=0.02  ^d^ p=0.004 |
| Leptin (ng/ml) | Male (n=2) | | 4.8  (2.2-11.2) | 31.0±11.1  [26.9(22.1-41.0)]^a,b^ | 6.9±5.8  [4.3(1.8-14.2)]^a,c^ | 32.4±10.7 [32.4(24.8-39.9)]^c^ | 2.1±0.2  [2.1(2.0-2.3)]^b^ | ^a*,b^ p<0.001  ^c^ p=0.05 |
|  | Female (n=6) | | 20.7  (3.9-77.3) | 60.6±16.5  [59.2(48.1-66.4)]^a,b^ | 12.5±9.2 [8.2(7.9-14.8)]^a,c^ | 59.4±9.2 [63.3(48.2-66.9)]^c,d^ | 12.7±10.6  [7.9(4.7-25.7)]^b,d^ | ^a*,b*,c,d^ p<0.001 |
| Resistin (ng/ml) | | | 13.8±4.6  (6.4-26.4) | 17.2±8.0  [15.5(12.9-20.7)] | 14.6±6.4 [12.8(9.9-20.5)] | 14.0±8.0 [9.9(8.0-20.5)] | 12.4±7.8  [9.8(9.0-11.8)] | NS |
| Cholesterol (mmol/l) | | | <5.0 | 5.2±0.9  [5.2(4.4-5.7)]^a,b^ | 4.6±0.7 [4.6(4.0-5.0)]^a^ | 5.1±1.1 [4.9(4.4-5.6)]^c^ | 4.3±0.3  [4.2(4.1-4.6)]^b,c^ | ^a^ p=0.03  ^b^ p=0.002  ^c^ p=0.04 |
| HDL-cholesterol (mmol/l) | | | >1.0 | 1.4±0.8  [1.2(1.1-1.6)]^a,b^ | 1.8±0.5 [1.8(1.6-2.3)]^a^ | 1.3±0.3  [1.3(1.0-1.4)]^c,d^ | 1.7±0.3  [1.8(1.3-2.0)]^b,c,d^ | ^a*^ p<0.001  ^b*,c^ p=0.02  ^d^ p=0.04 |
| LDL-cholesterol (mmol/l) | | | <3.0 | 3.5±1.1  [3.1(2.8-4.1)]^a,b^ | 2.7±0.5 [2.7(2.2-3.1)]^a,c^ | 3.3±0.8 [3.0(2.8-4.1)]^c,d^ | 2.5±0.4  [2.5(2.1-2.9)]^b,d^ | ^a^ p=0.005  ^b^ p=0.03  ^c^ p=0.01  ^d^ p=0.02 |
| Triglycerides (mmol/l) | | | <1.7 | 1.9±1.0  [1.9(1.2-2.1)]^a,b^ | 1.1±0.5 [0.9(0.7-1.5)]^a,c^ | 1.7±0.7 1.5[(1.2-2.4)]^c^ | 1.2±0.4  [1.1(0.9-1.5)]^b^ | a* p<0.001  ^b, c*^ p=0.03 |
| hs-CRP (mg/l) | | | <5.0 | 6.6±6.2  [4.0(2.0-12.0)]^a,b^ | 1.2±0.6 [1.0(1.0-1.0)]^a,c^ | 4.1±1.7 [4.0(2.3-6.0)]^b,c,d^ | 1.3±0.5  [1.0(1.0-1.8)]^d^ | ^a,b*,c*,d*^ p<0.001 |
| Glucose (mmol/l) | | | 4.1-6.1 | 7.0±2.7  [5.9(5.3-8.1)]^a,b^ | 5.4±1.3 [5.1(4.8-5.7)]^a,c^ | 6.0±0.6 [6.1(5.6-6.6)]^c,d^ | 4.6±0.8  [4.8(4.4-5.2)]^b,d^ | ^a*^ p=0.005  ^b*,d^ p=0.003  ^c*^ p=0.02 |
| A1C (%) | | | 4.0-6.0 | 6.2±1.4  [5.7(5.5-6.2)]^a^ | 5.4±0.4  [5.3(5.1-5.6)]^a^ | 5.6±0.3  [5.7(5.3-5.8)]^b^ | 5.2±0.4  [5.2(5.0-5.4)]^b^ | ^a^ p=0.01  ^b^ p=0.03 |
| A1C (IFCC) (mmol/l) | | | 20.0-42.0 | 44.0±14.8  [39.0(37.0-43.3)]^a,b^ | 35.0±4.9  [34.0(32.0-37.5)]^a^ | 37.6±3.0 [38.5(34.5-40.0)]^c^ | 33.3±4.1 [33.0(30.5-35.5)]^b,c^ | ^a*^ p<0.001  ^b^ p=0.002  ^c^ p=0.03 |
| Vitamin D (25-OH) (nmol/l) | | | ≥ 75.0 | 55.±12.02  [57.9(44.6-63.0)]^a^ | 68.4±23.0 [73.3(47.4-80.8)]^a^ | 51.1±21.3 [46.8(34.0-65.2)] | 66.6±18.3 [64.9(51.5-84.2)] | ^a^ p=0.02 |
| Vitamin B12 (pmol/l) | | | 145.0-569.0 | 303.0±121.2  [290.5(232.8-359.2)] | 304.9±115.3  [280.5(213.0-389.8)] | 308.3±65.4  [323.0(242.3-349.0)] | 275.3±137.3  [239.0(163.0-316.0)] | NS |
| Vitamin B9  (nmol/l) | | | 8.8-60.8 | 15.7±8.0  [15.0(9.9-20.3)]^a^ | 30.2±23.5  [24.4(10.5-38.9)]^a^ | 14.2±6.2  [12.5(11.2-20.2)] | 24.9±25.4  [13.6(11.9-25.3)] | ^a^p=0.01 |
| Ferritin (µg/l) | | Male (n=9) | 30.0–400.0 | 187.5±84.7  [221.3(103.5-257.4)] | 171.9±70.8  [160.6(112.9-235.2)] | 268.7±19.9  [19.9(268.7-282.8)] | 286.8±135.5  [286.8(191.0-382.6)] | ND* |
|  |  | Female  (n=13) | 13.0–150.0 | 103.4±67.4  [84.0(60.0-143.3)] | 95.5±76.0  [76.9(26.3-186.9)] | 125.3±68.5  [124.1(59.9-183.3)] | 162.6±113.2  [166.3(51.6-271.9)] | NS |
| Fe  (µmol/l) | | Male  (n=9) | 10.6-28.3 | 19.0±5.7  [17.8(14.1-24.3)] | 21.6±7.2  [19.1(17.1-27.2)] | 19.7±8.1  [19.7(13.9-25.4)] | 25.1±0.1  [25.1(25.0-25.1)] | ND* |
|  |  | Female  (n=13) | 6.6-26.0 | 15.2±5.3  [13.0(12.4-17.4)] | 20.9±9.8  [19.4(16.2-24.8)] | 18.3±7.5  [18.2(13.0-22.2)] | 19.7±8.9  [18.9(12.6-27.1)] | NS |

*Mann-Whitney Rank Sum Test; ** t-test; NS-not significant, ND*-not detected due to small amounts of patients; A1C – glycated hemoglobin; Fe-iron.
